# Supplementary material for: Micrometastasis-derived models enable drug testing for early-stage, high-risk melanoma patients
Source: EMBO Mol Med. 2025 Dec 5;18(1):297–324. doi: 10.1038/s44321-025-00339-8 (PMC12808144; doi:10.1038/s44321-025-00339-8)
Supplement: Supplementary file 1 — Appendix [file 44321_2025_339_MOESM1_ESM.pdf]

Appendix

Micrometastasis-derived models enable drug testing for early-stage, high-risk melanoma patients

Kathrin Weidele, Christian Werno, Steffi Treitschke, Catherine Botteron, Martin Hoffmann, Sebastian Scheitler, Lukas Wöhrl, Zbigniew Czyz, Giancarlo Feliciello, Florian Weber, Adithi Ravikumar Varadarajan, Jens Warfsmann, Silvia Materna-Reichelt, Marie Katzer, Laura Schreieder, Parvaneh Mohammadi, Hedayatollah Hosseini, Kamran Honarnejad, Sebastian Haferkamp, Melanie Werner-Klein and Christoph A. Klein

Table of content

Appendix Figure S1: Histological characterization of patient and PDX samples page 1

Appendix Figure S2: Genome-wide copy number alteration (CNA) profiles page 2-3

Appendix Figure S3: **:** Comparison of CLs generated by approach I and II page 4

Appendix Figure S1

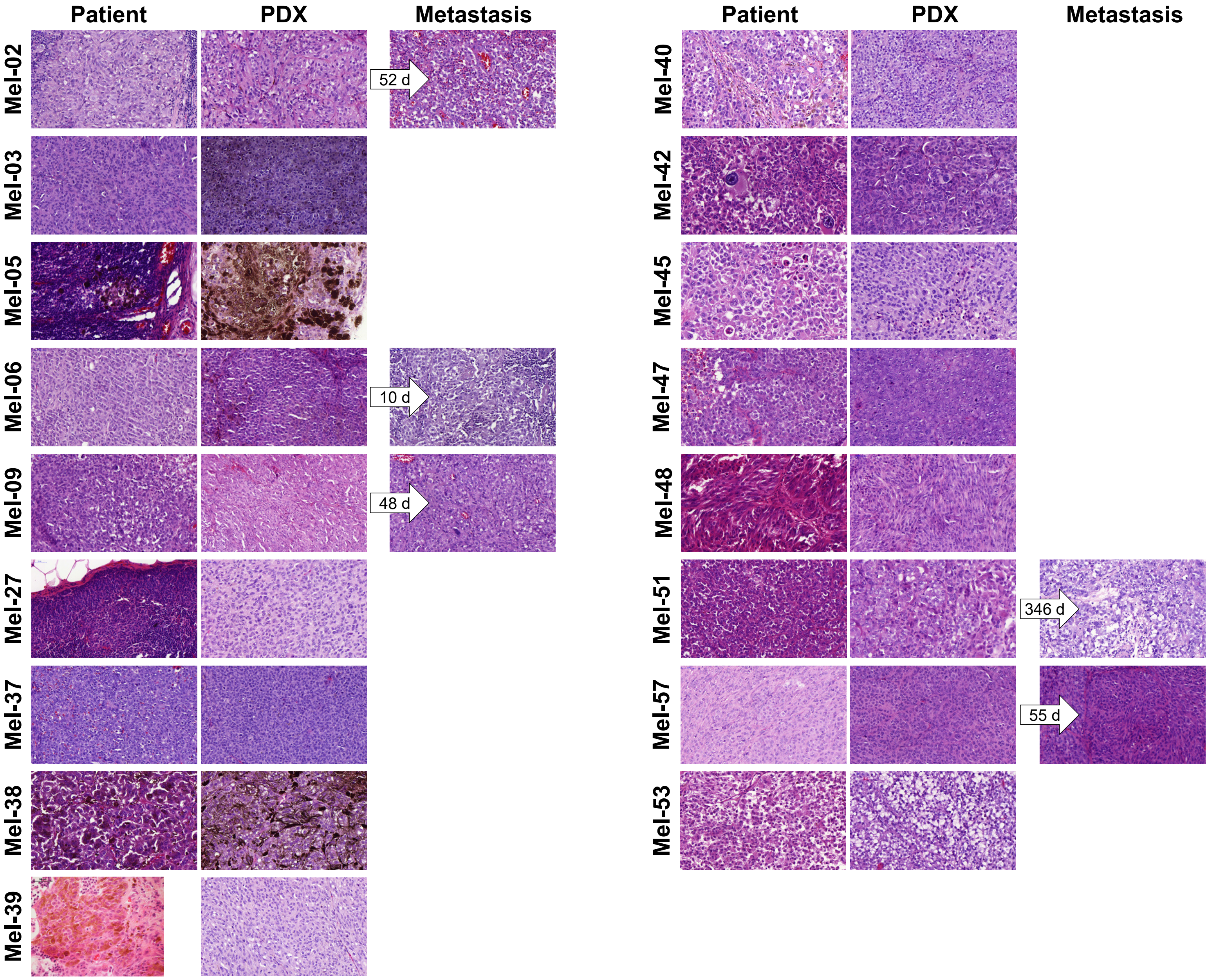

Appendix Figure S1: Histological characterization of patient and PDX samples
Histological side by side comparison of matching samples of representative patients detected by routine pathology using H&E staining of one half of the LN with the corresponding xenograft tumors and, if available, later arising metastasis.

Appendix Figure S2

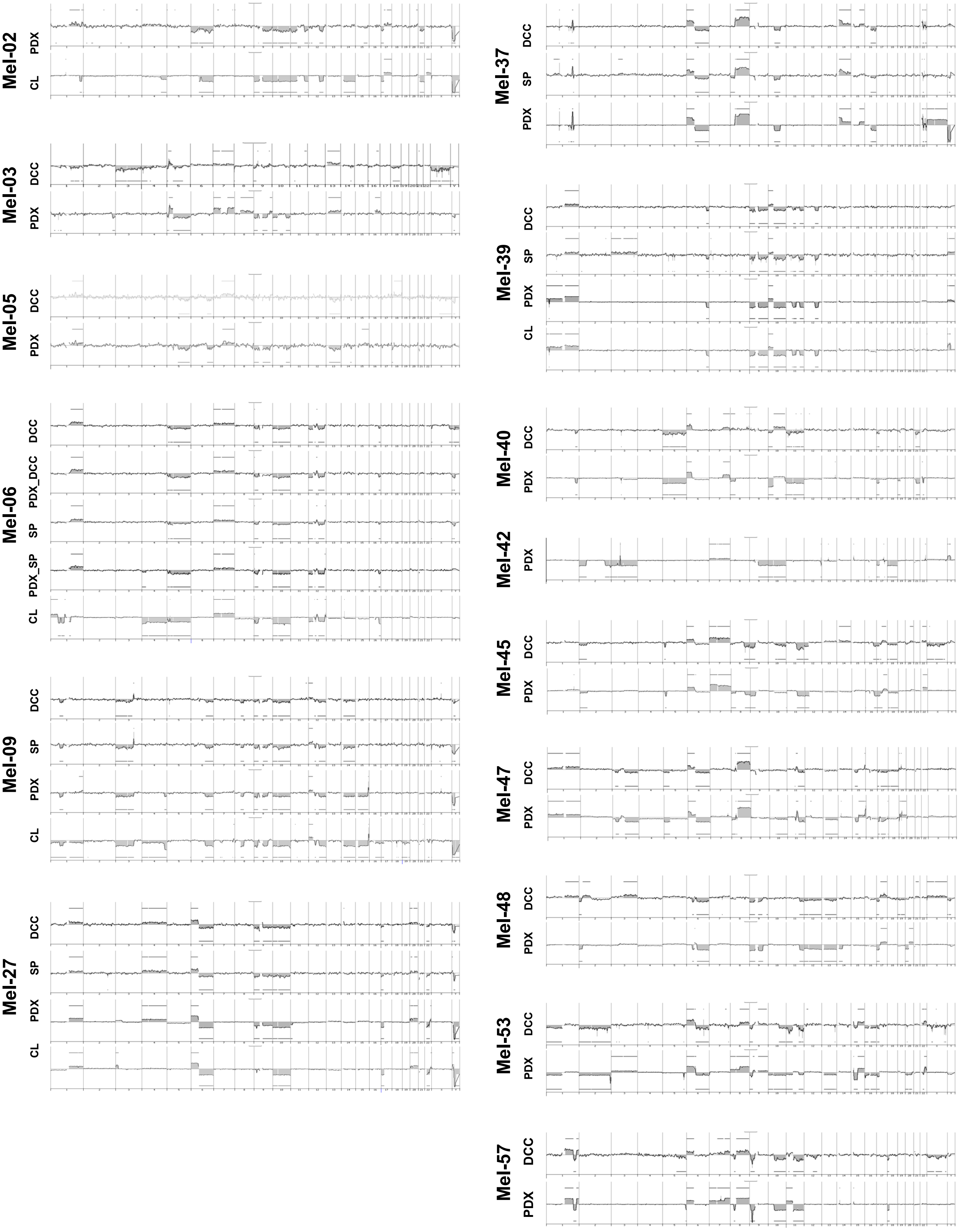

Appendix Figure S2

Appendix Figure S2: Genome-wide copy number alteration (CNA) profiles
CNA analyses of 15 patients, where we established DCC-derived xenograft models (PDX) from 1 lymph node (LN). PDX and, if available, matched pairs of DCCs, DCC-derived spheres (SP) or *in vitro* generated cell lines (CL) are shown. Histogram of genomic gains and losses are shown.

Appendix Figure S3

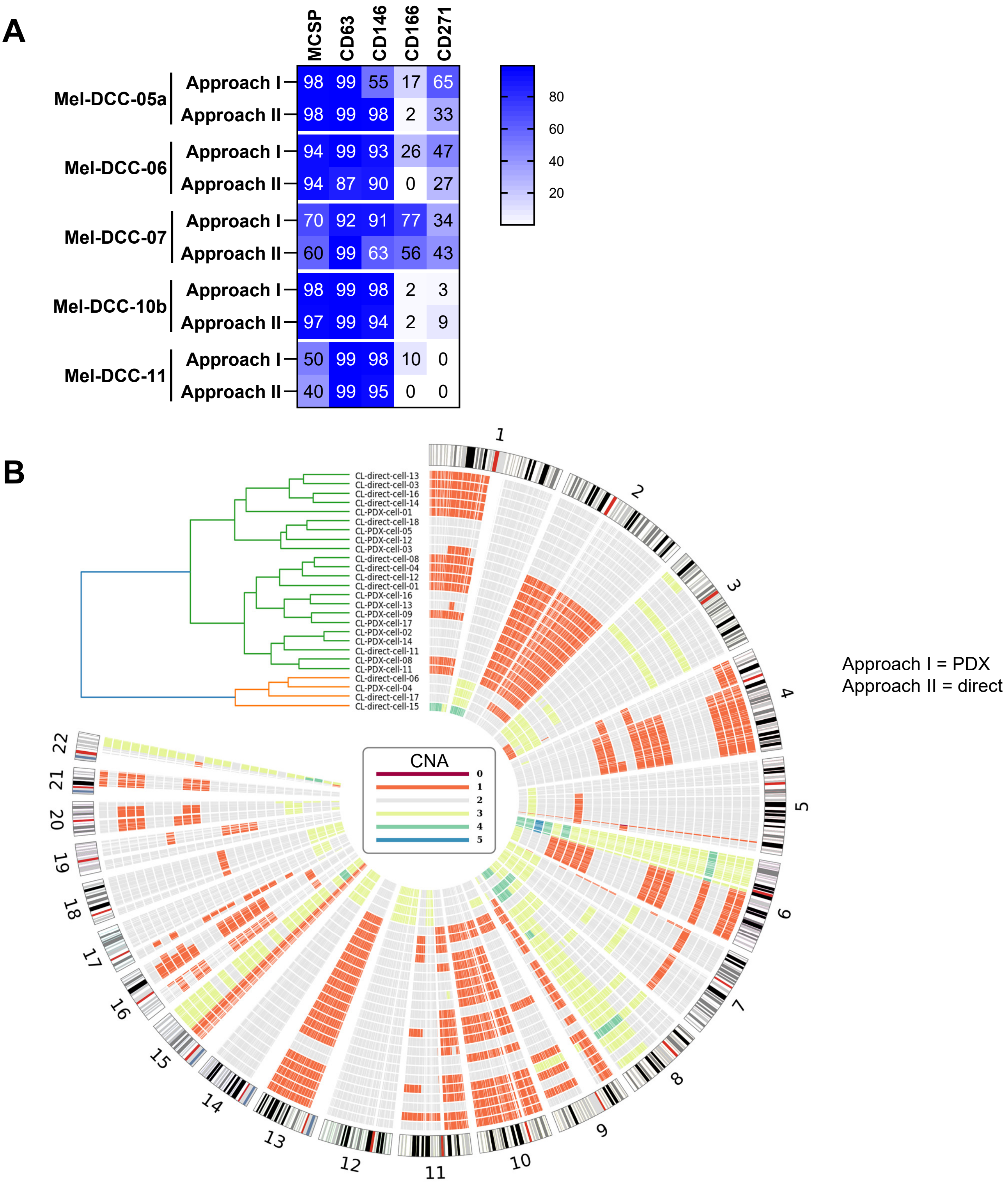

Appendix Figure S3: Comparison of CLs generated by approach I and II

A Flow cytometry analysis of melanoma marker expression (MCSP, CD63, CD146, CD166, CD271). The scores (color scale) indicate the percentage of marker-positive cells compared to isotype controls.

B Circos plot. Comparison of CNA profiles of individual isolated cells from CLs derived from patient Mel-53 generated both via expansion approach I ('PDX', n = 13 single cells) and approach II ('direct', n = 13 single cells). Concentric circular tracks represent the autosomal CNA profiles. Hierarchical clustering was performed using Ward's method. The color coding of the copy numbers is given in the central legend of the figure. The copy number indicates how many copies of a segment are present: Based on the underlying assumption that diploidy was originally present, 0 stands for the complete loss of both alleles, 1 for the loss of one copy, 2 corresponds to the normal (diploid) state, and 3 and higher values indicate an increase in copies up to multiple duplications. Samples processed with low-pass whole genome sequencing.
