# Supplementary figures and images for: Micrometastasis-derived models enable drug testing for early-stage, high-risk melanoma patients
Source: EMBO Mol Med. 2025 Dec 5;18(1):297–324. doi: 10.1038/s44321-025-00339-8 (PMC12808144; doi:10.1038/s44321-025-00339-8)

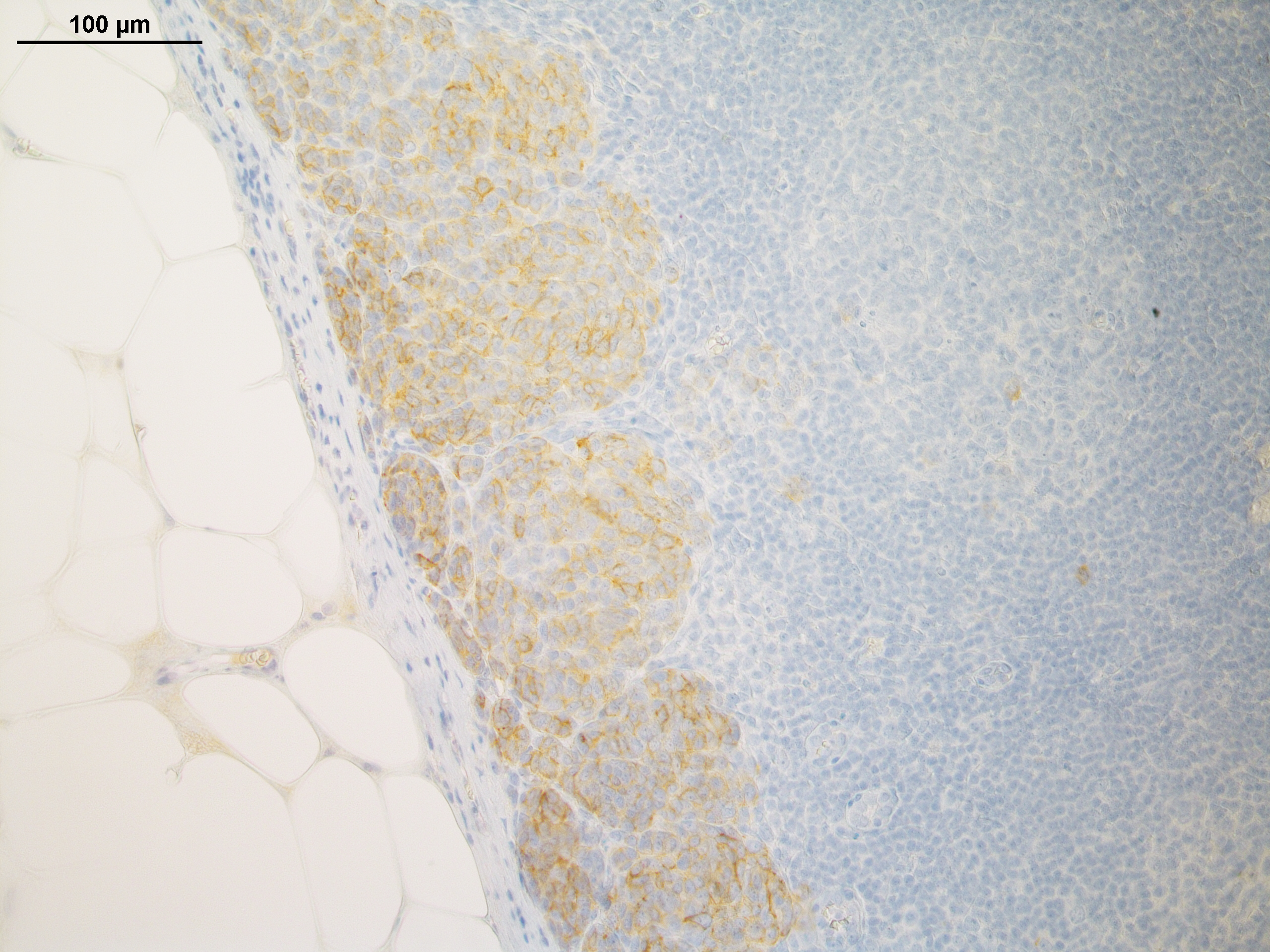

Supplement: Supplementary file 5 — Source data Fig. 2 [file 44321_2025_339_MOESM5_ESM.zip › Figure 2/2A/Mel-27-Patient LN.jpg]

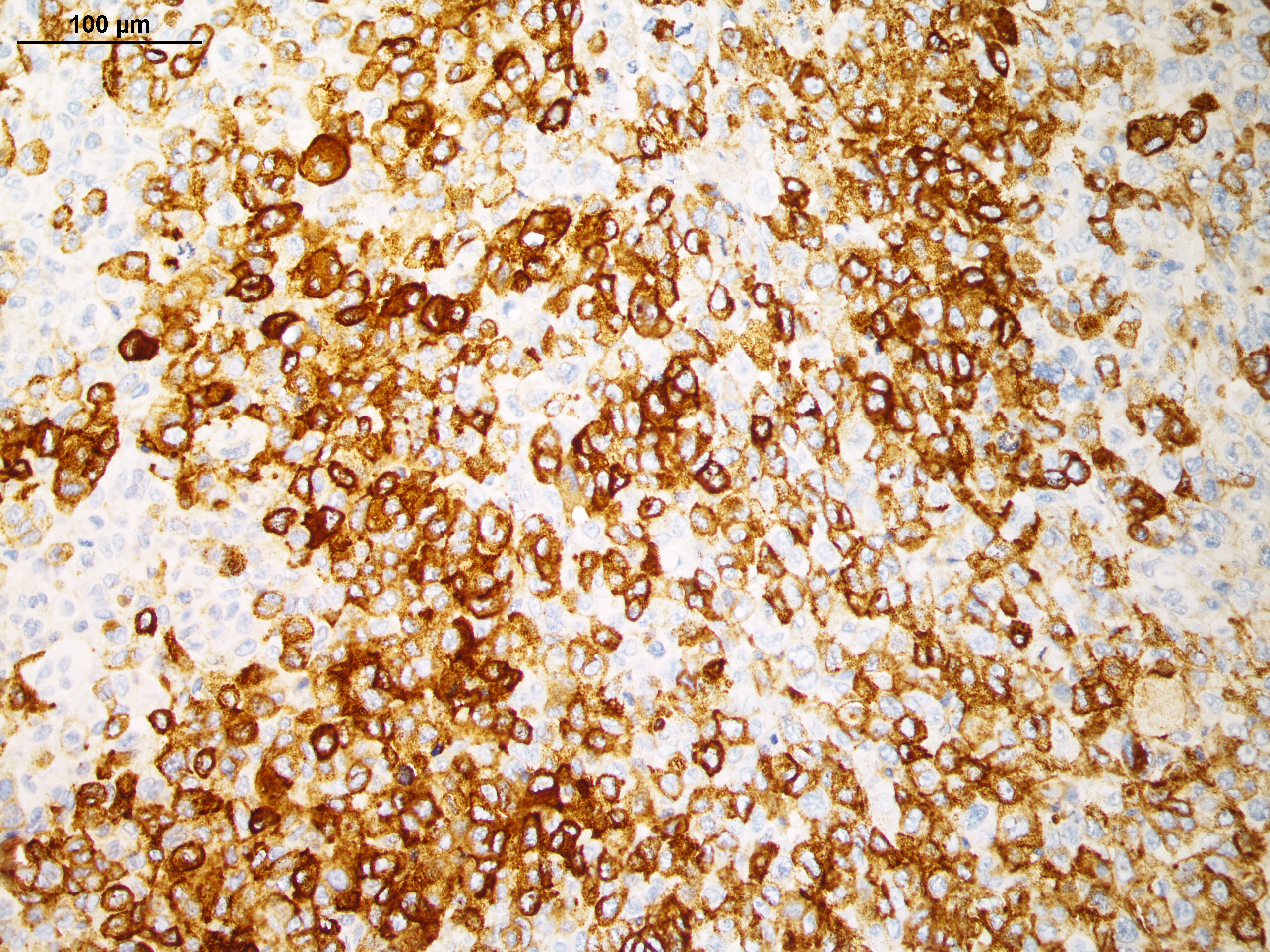

Supplement: Supplementary file 5 — Source data Fig. 2 [file 44321_2025_339_MOESM5_ESM.zip › Figure 2/2A/Mel-27-PDX.jpg]

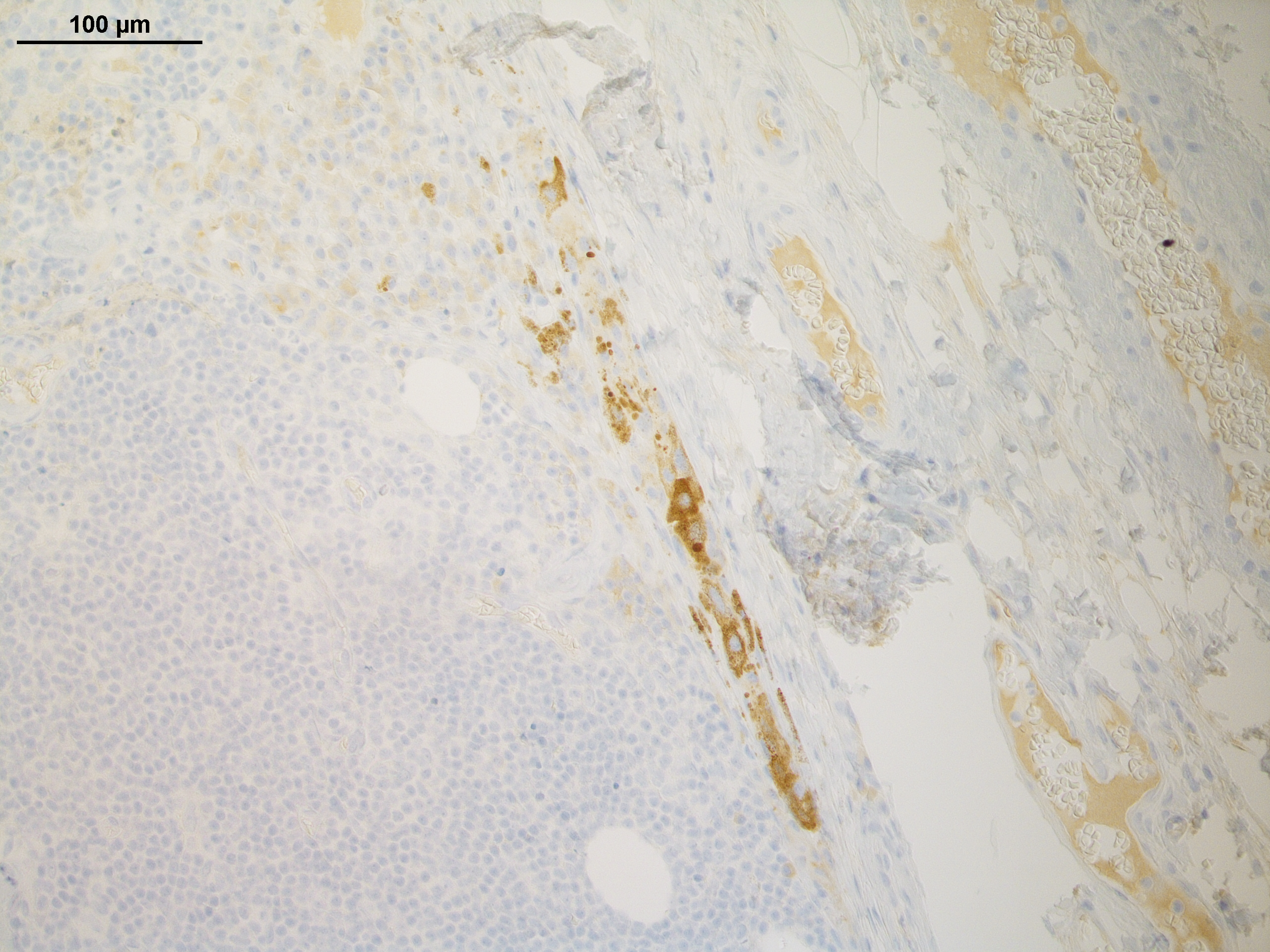

Supplement: Supplementary file 5 — Source data Fig. 2 [file 44321_2025_339_MOESM5_ESM.zip › Figure 2/2A/Mel-39-Patient LN.jpg]

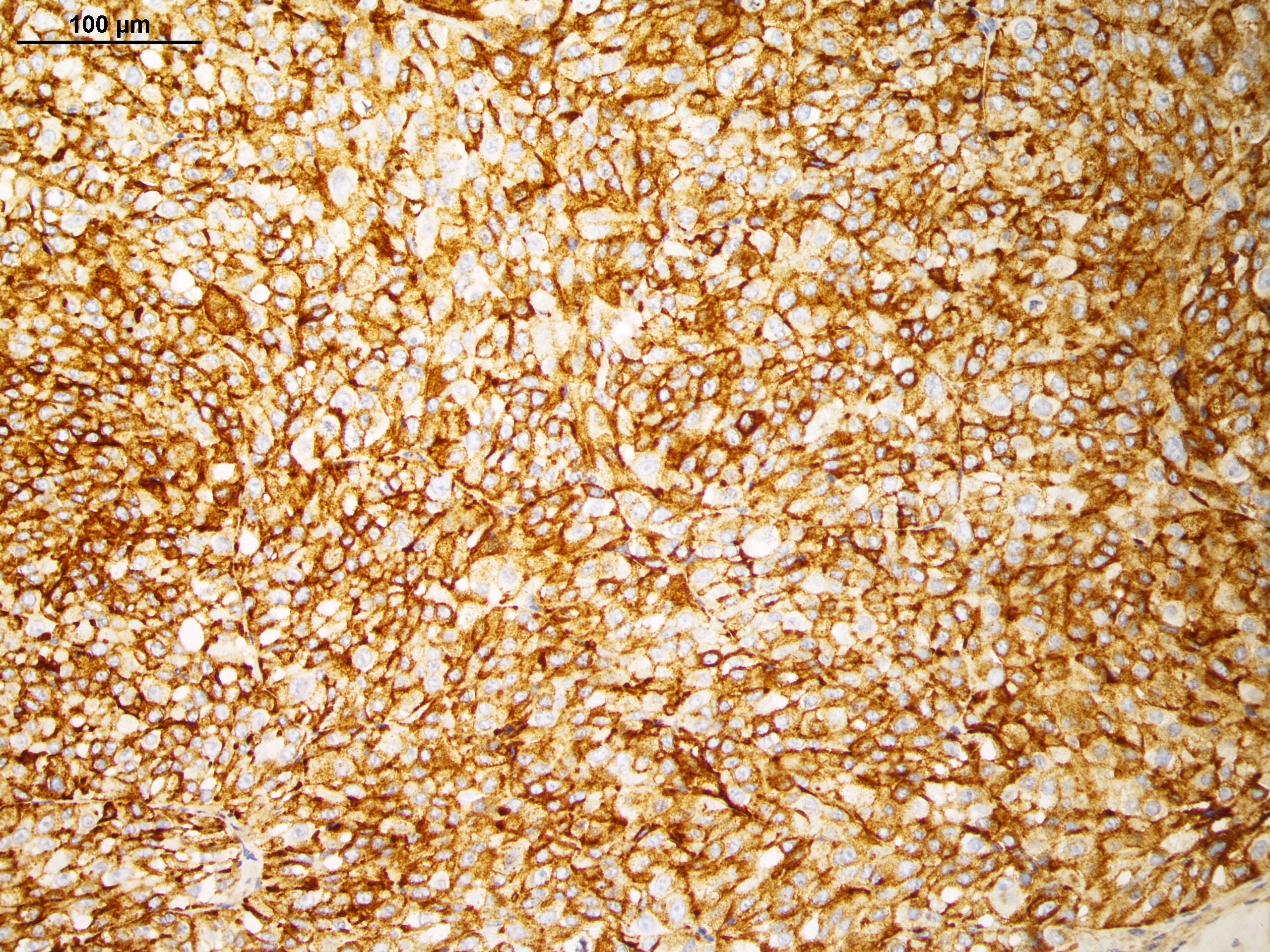

Supplement: Supplementary file 5 — Source data Fig. 2 [file 44321_2025_339_MOESM5_ESM.zip › Figure 2/2A/Mel-39-PDX.jpg]

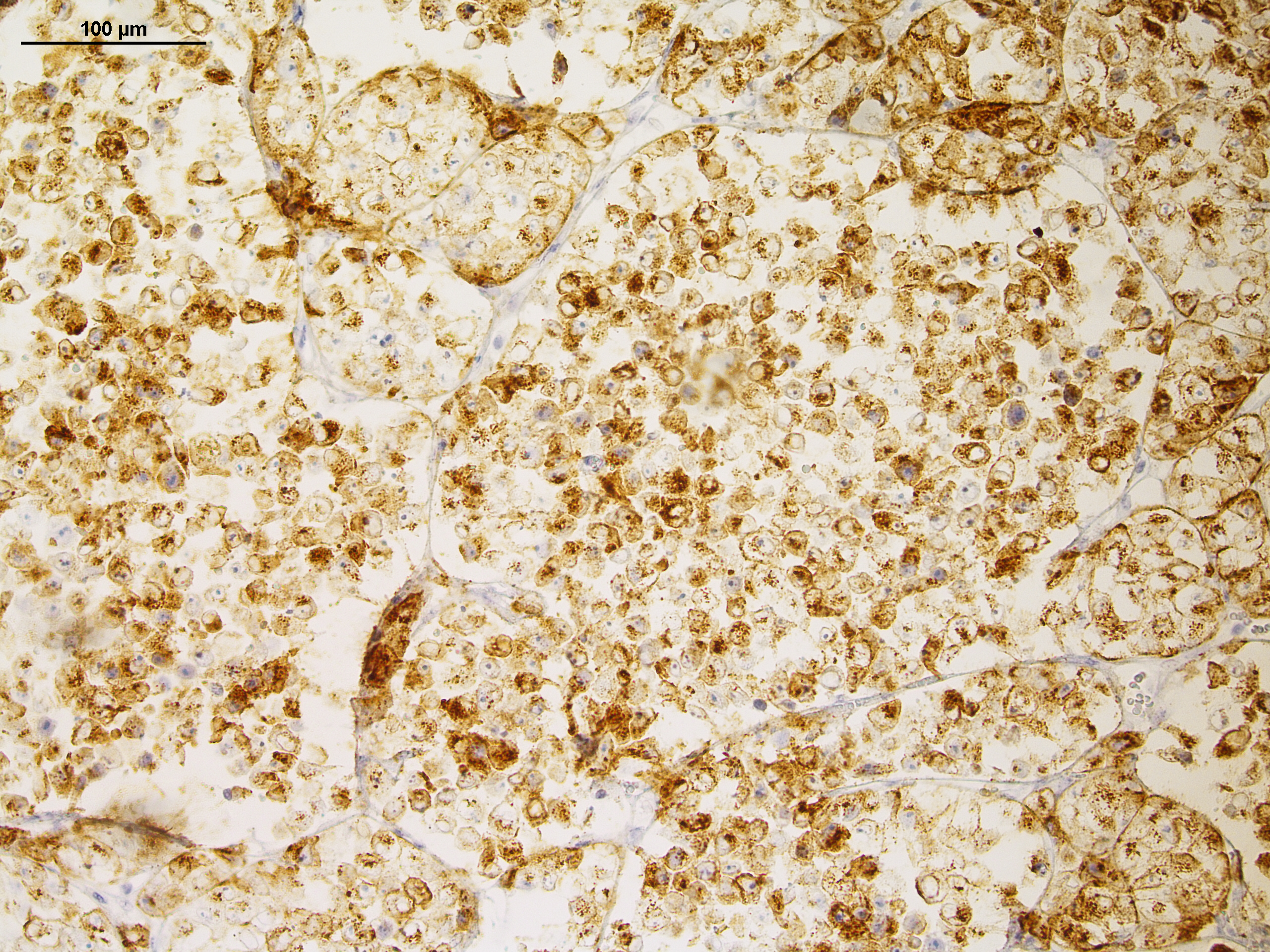

Supplement: Supplementary file 5 — Source data Fig. 2 [file 44321_2025_339_MOESM5_ESM.zip › Figure 2/2A/Mel-53-PDX.jpg]

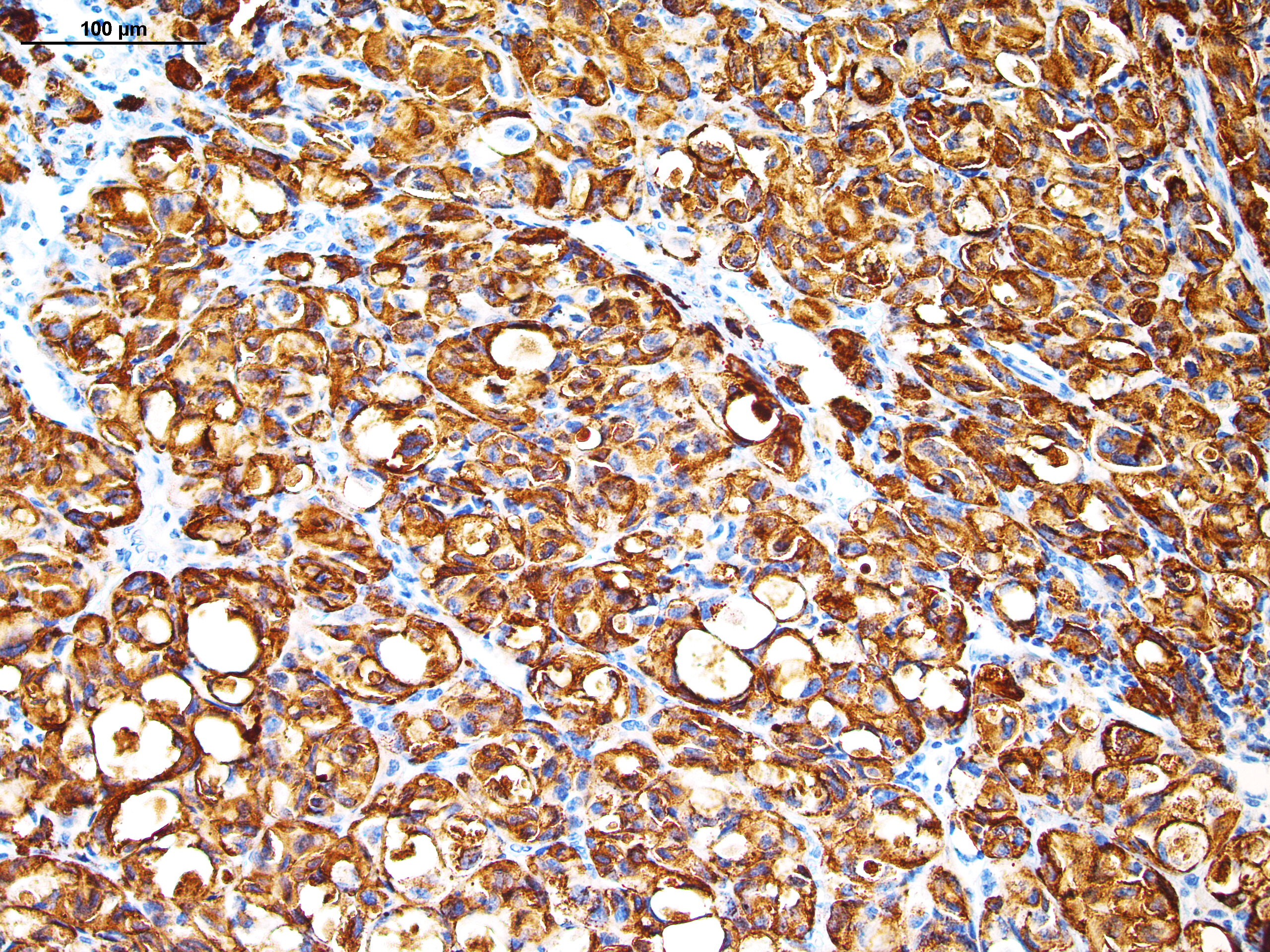

Supplement: Supplementary file 5 — Source data Fig. 2 [file 44321_2025_339_MOESM5_ESM.zip › Figure 2/2A/Mel-53_Patient LN.jpg]

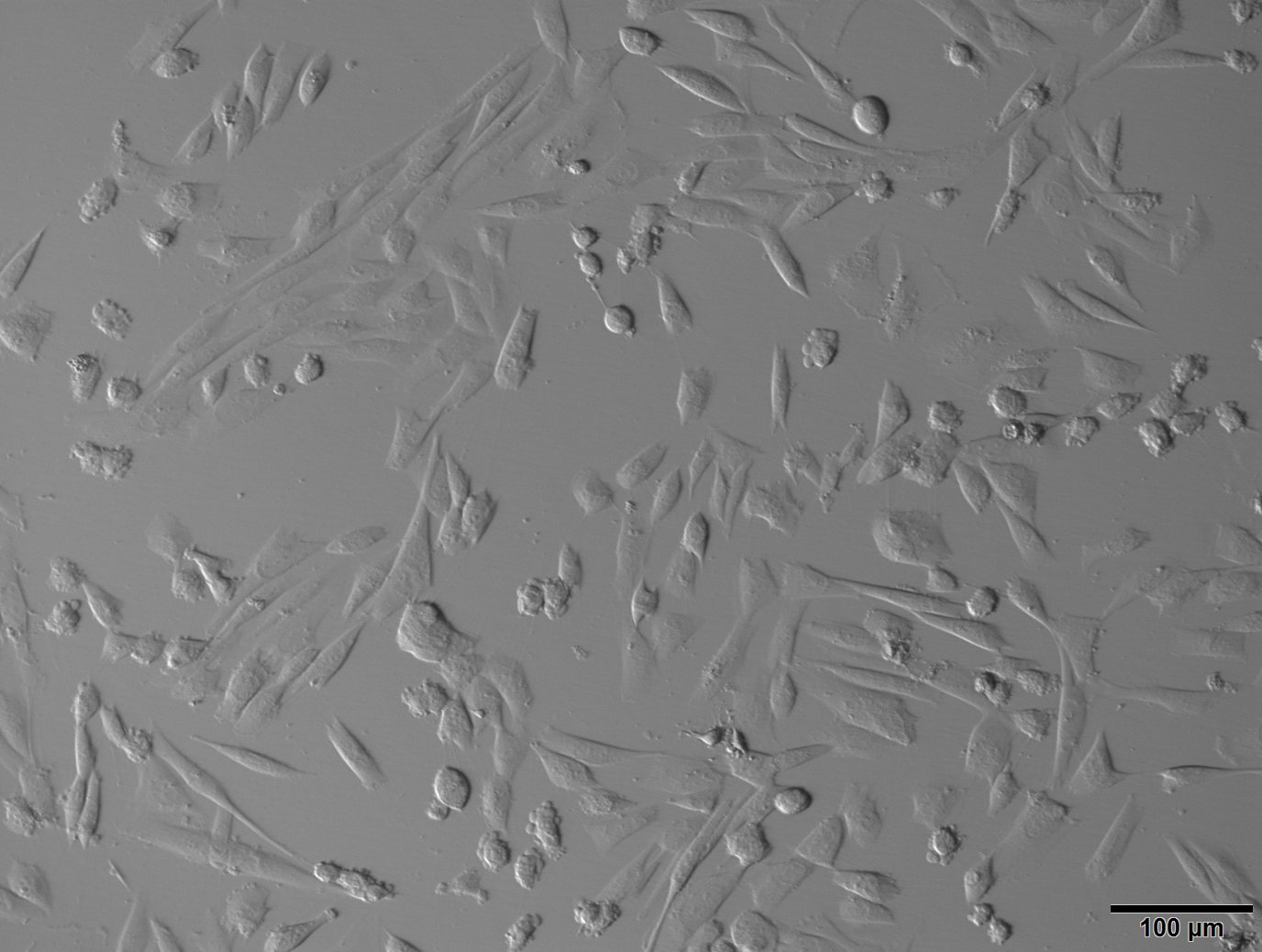

Supplement: Supplementary file 5 — Source data Fig. 2 [file 44321_2025_339_MOESM5_ESM.zip › Figure 2/2A/Mel-DCC-04_10X_.jpg]

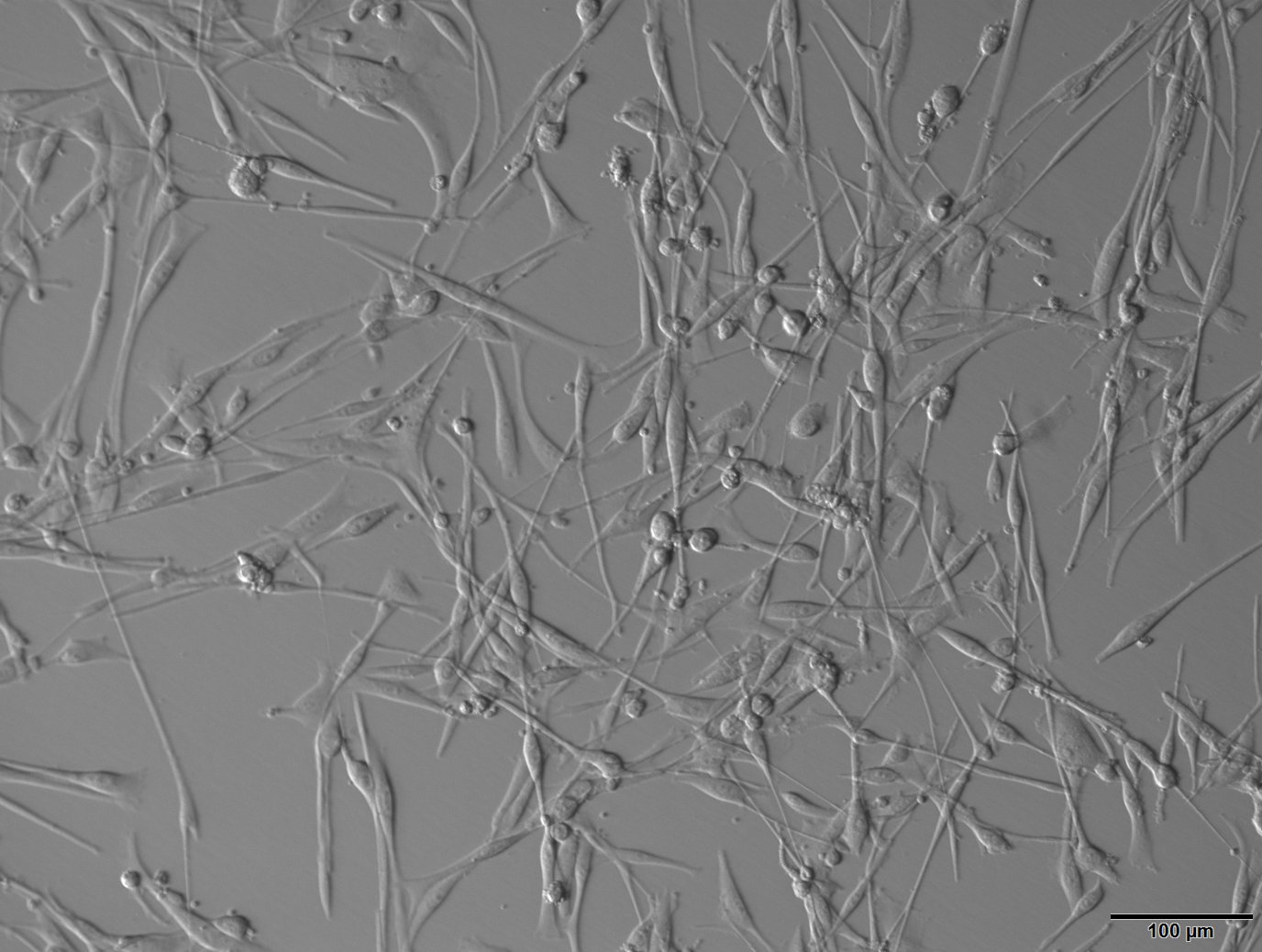

Supplement: Supplementary file 5 — Source data Fig. 2 [file 44321_2025_339_MOESM5_ESM.zip › Figure 2/2A/Mel-DCC-09_10X.jpg]

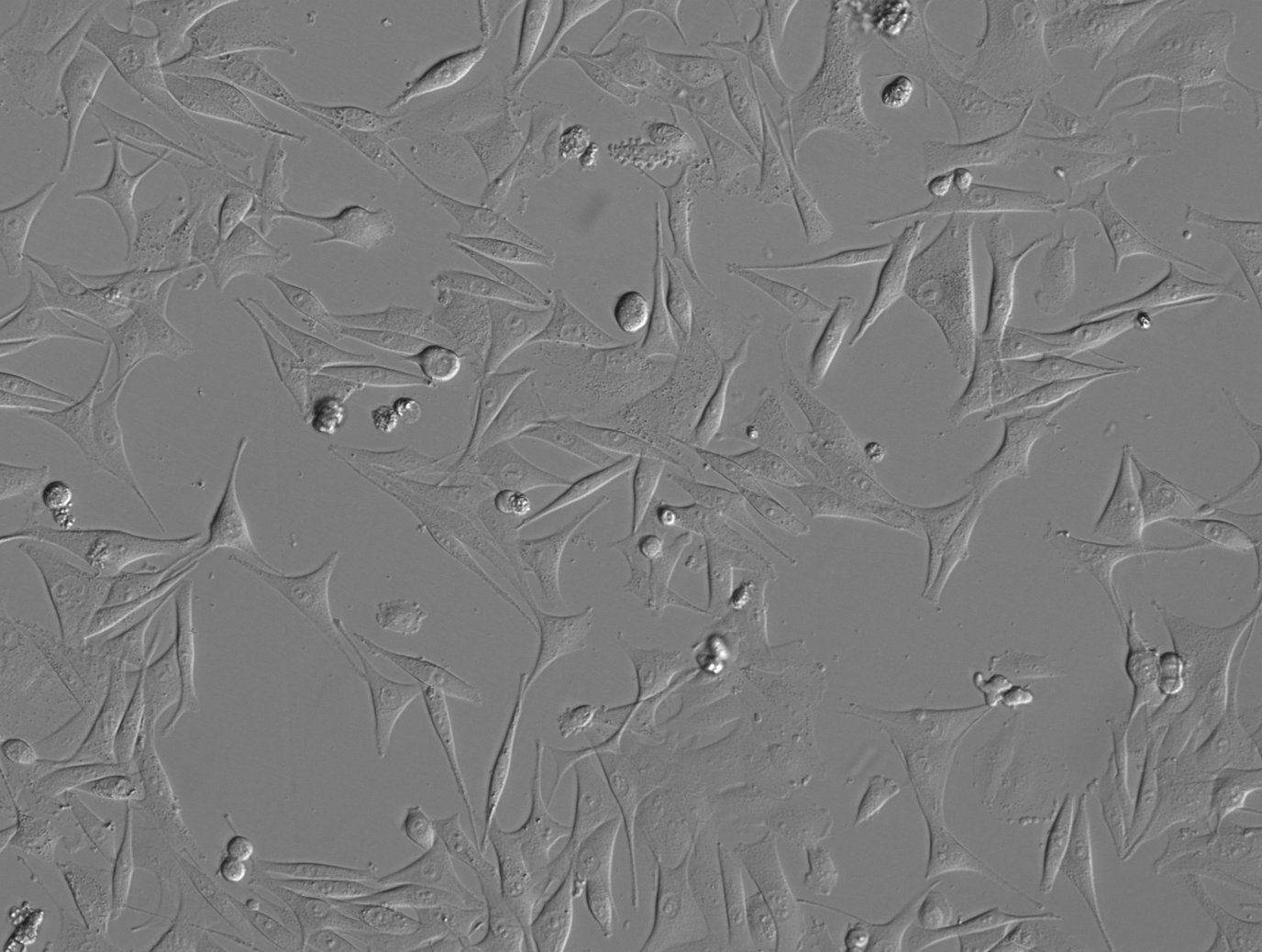

Supplement: Supplementary file 5 — Source data Fig. 2 [file 44321_2025_339_MOESM5_ESM.zip › Figure 2/2A/Mel-DCC-11_10X.jpg]

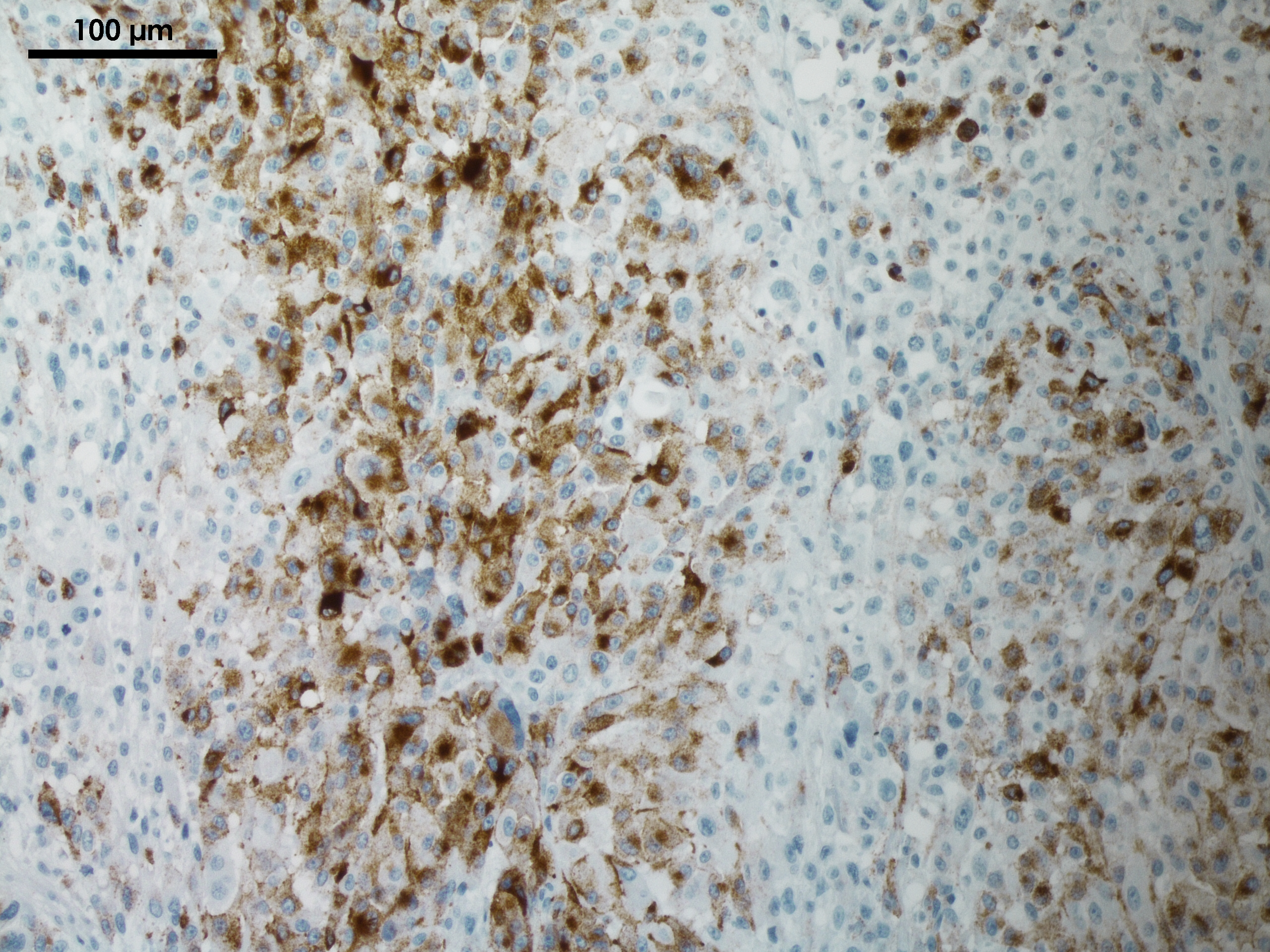

Supplement: Supplementary file 5 — Source data Fig. 2 [file 44321_2025_339_MOESM5_ESM.zip › Figure 2/2C/Mel-09-Met.jpg]

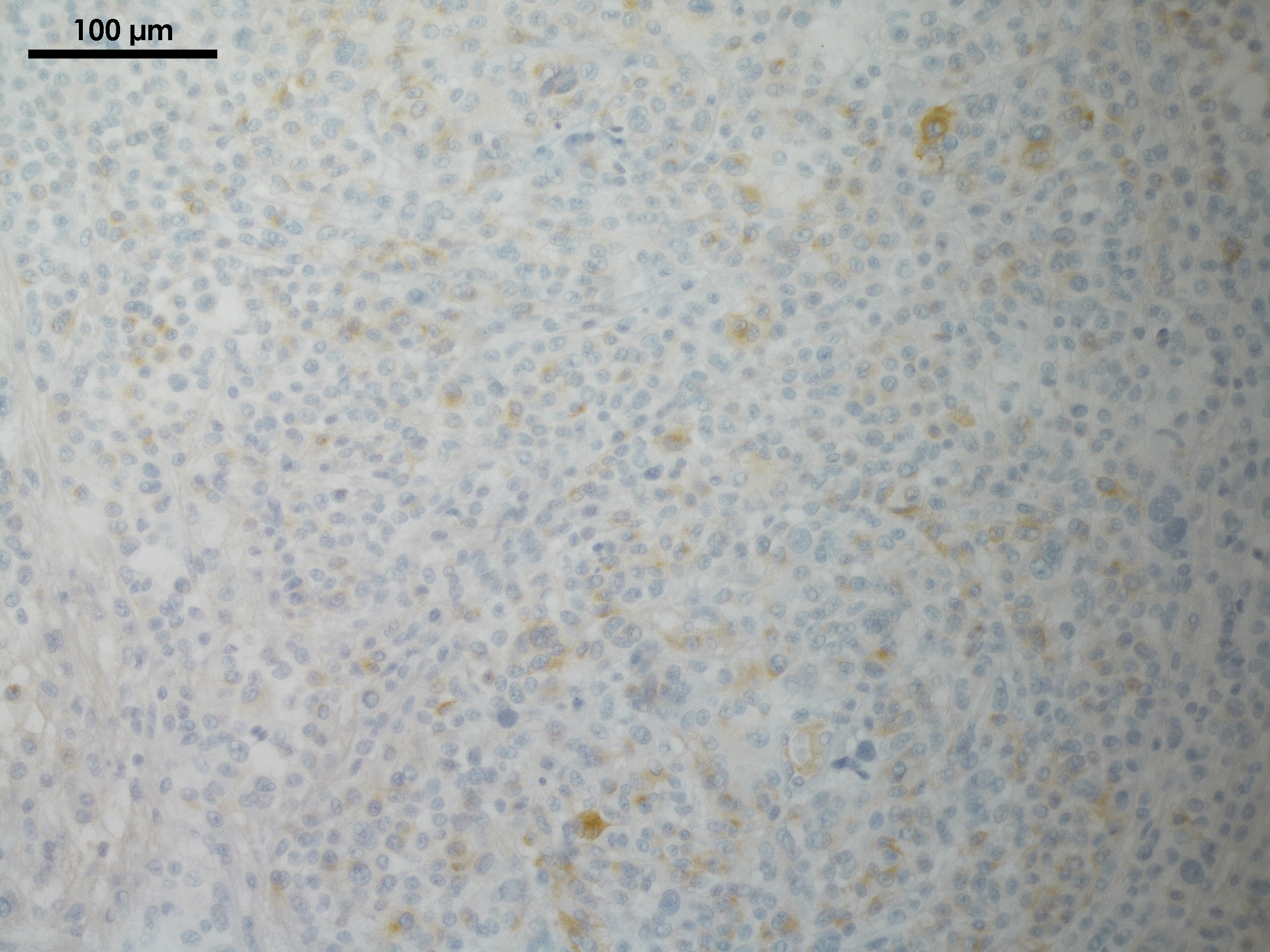

Supplement: Supplementary file 5 — Source data Fig. 2 [file 44321_2025_339_MOESM5_ESM.zip › Figure 2/2C/Mel-09-Patient LN.jpg]

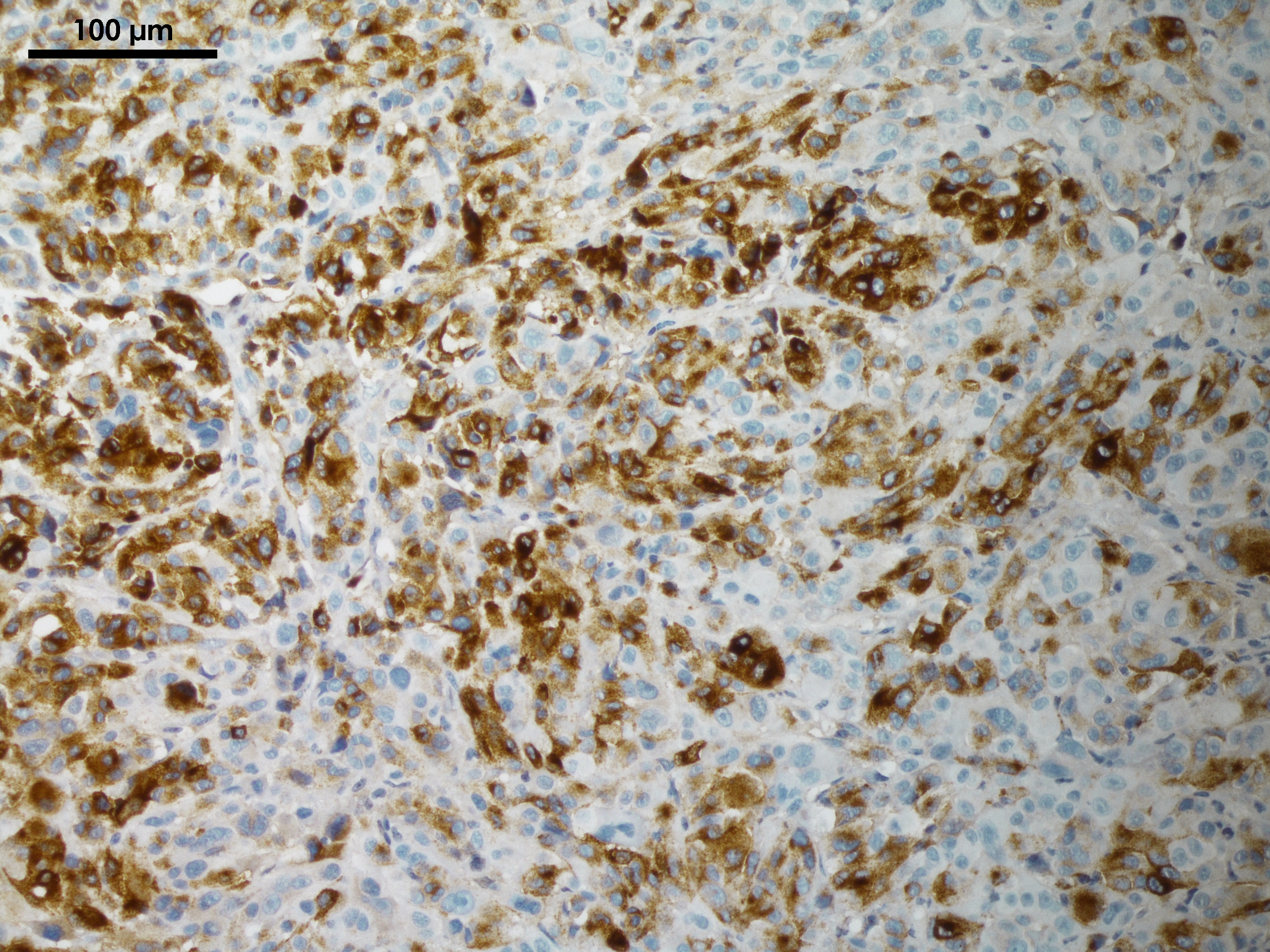

Supplement: Supplementary file 5 — Source data Fig. 2 [file 44321_2025_339_MOESM5_ESM.zip › Figure 2/2C/Mel-09-PDX.jpg]

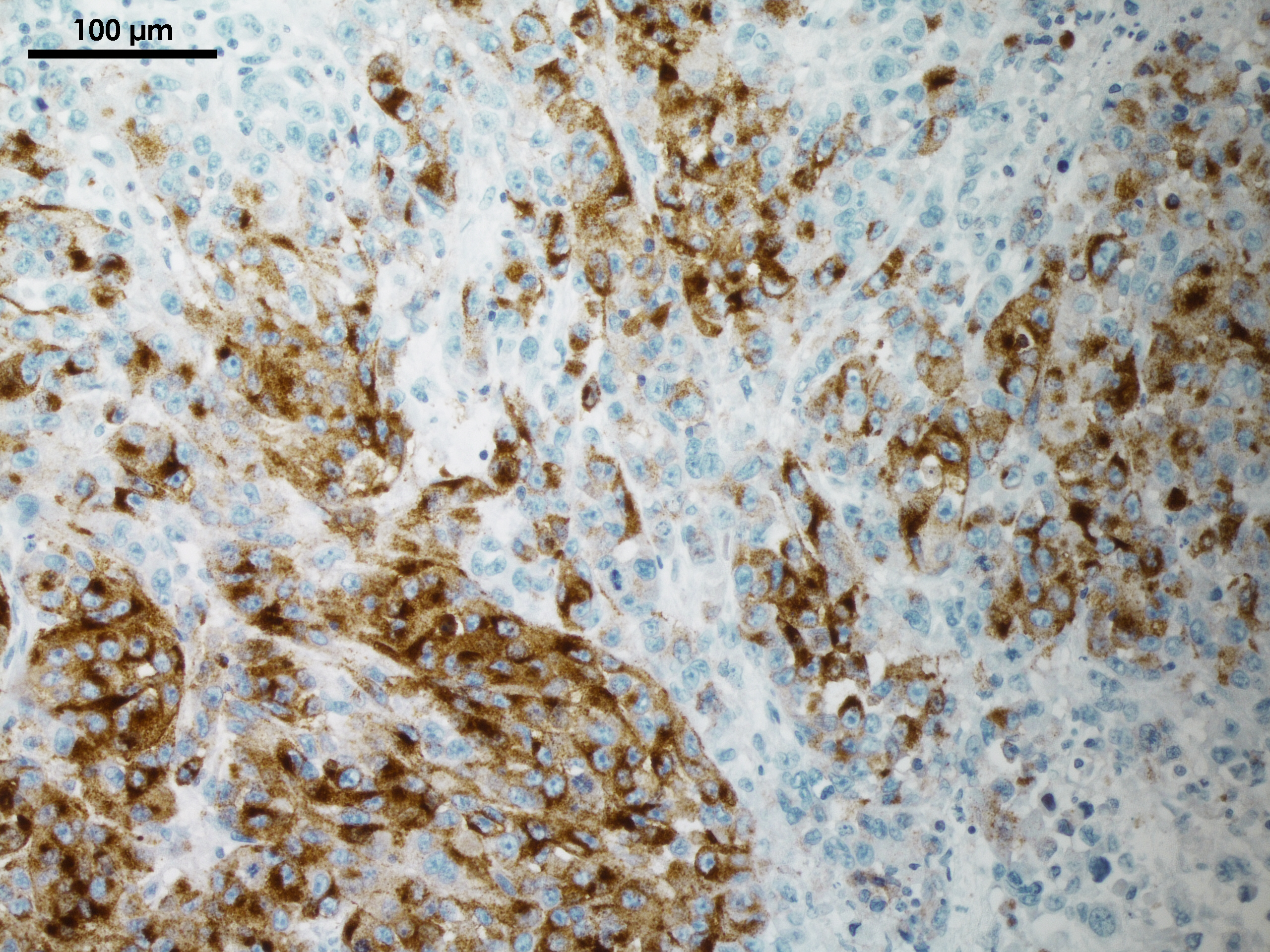

Supplement: Supplementary file 5 — Source data Fig. 2 [file 44321_2025_339_MOESM5_ESM.zip › Figure 2/2C/Mel-51-Met.jpg]

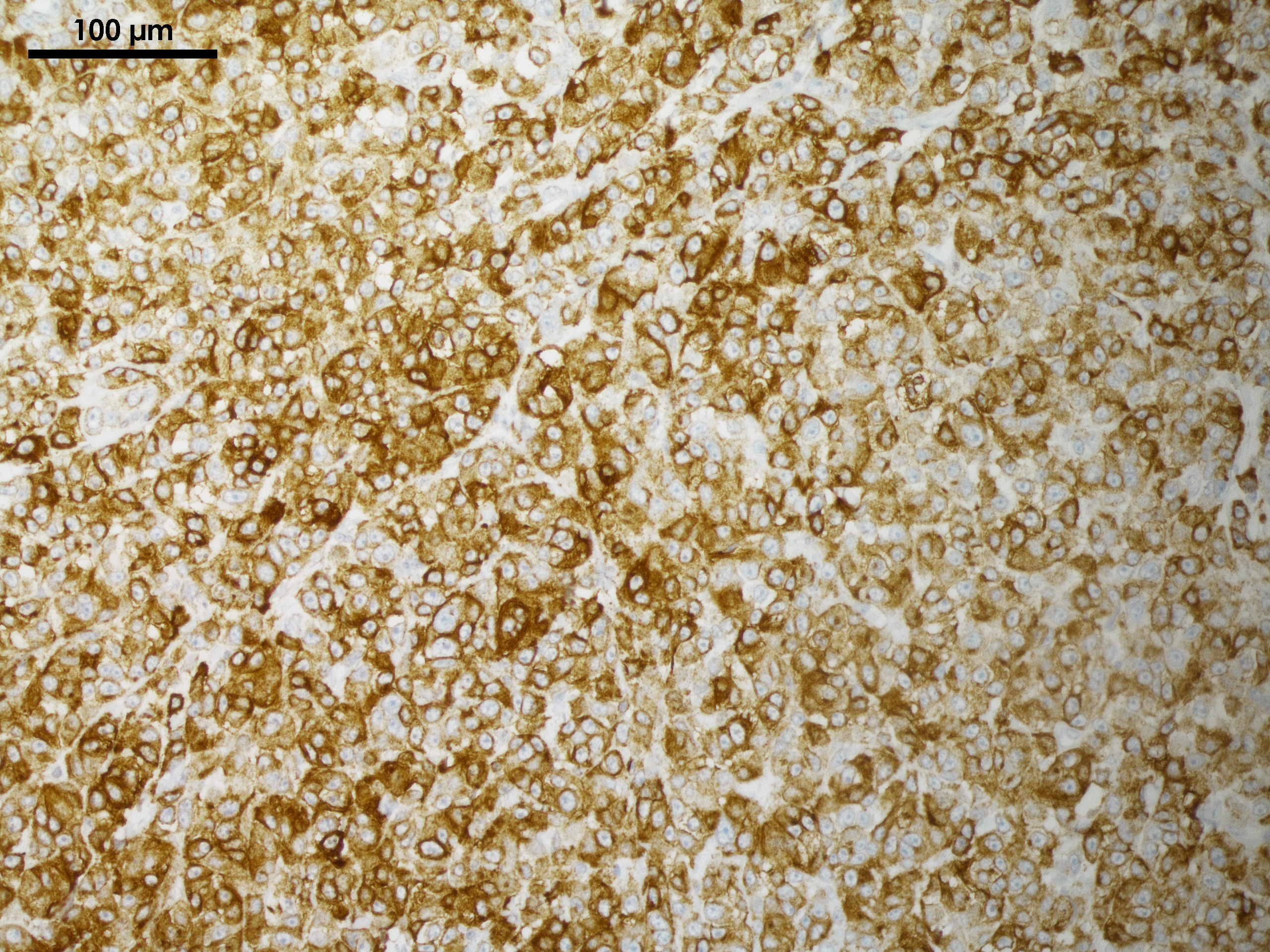

Supplement: Supplementary file 5 — Source data Fig. 2 [file 44321_2025_339_MOESM5_ESM.zip › Figure 2/2C/Mel-51-Patient LN.jpg]

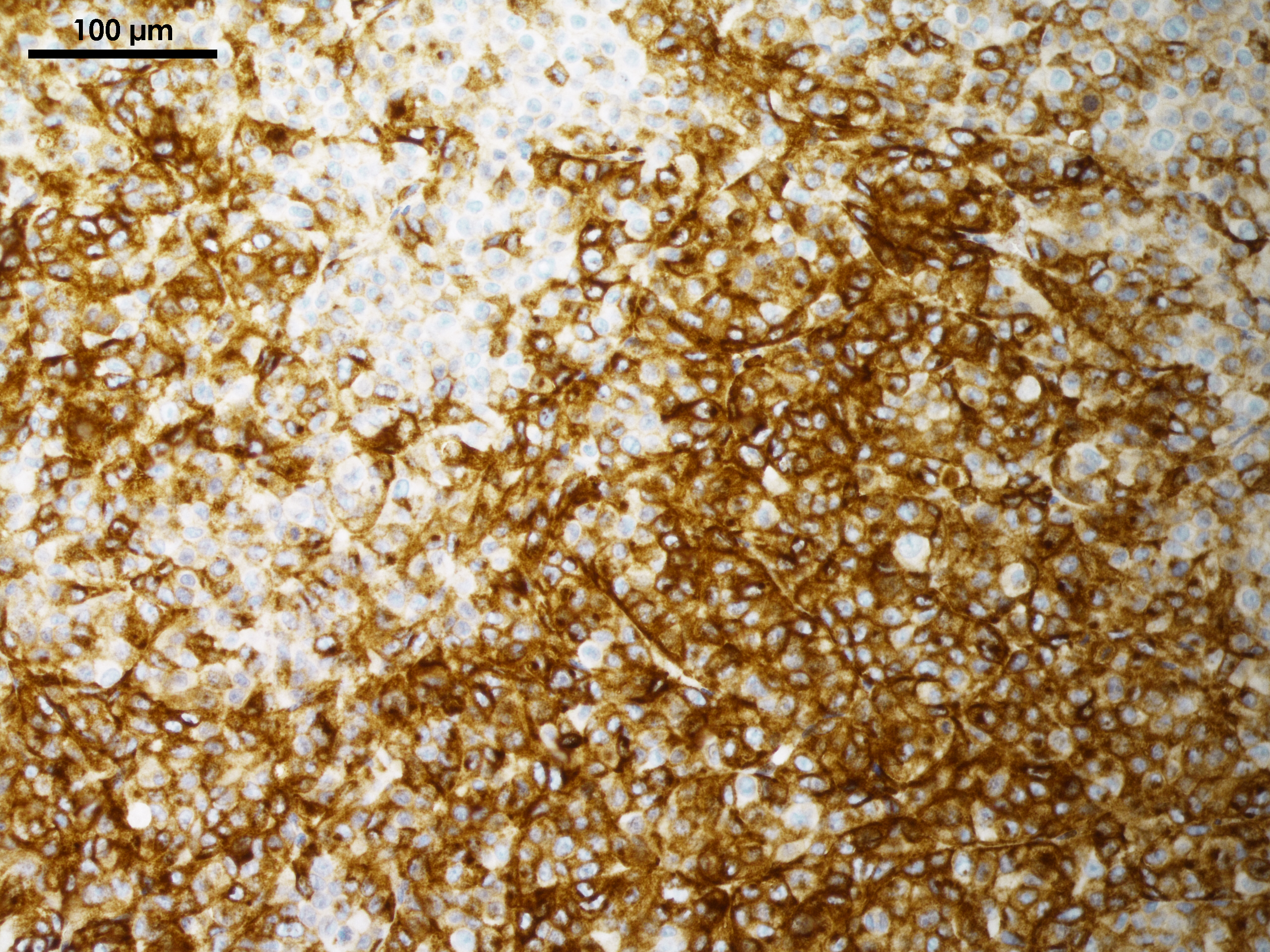

Supplement: Supplementary file 5 — Source data Fig. 2 [file 44321_2025_339_MOESM5_ESM.zip › Figure 2/2C/Mel-51-PDX.jpg]

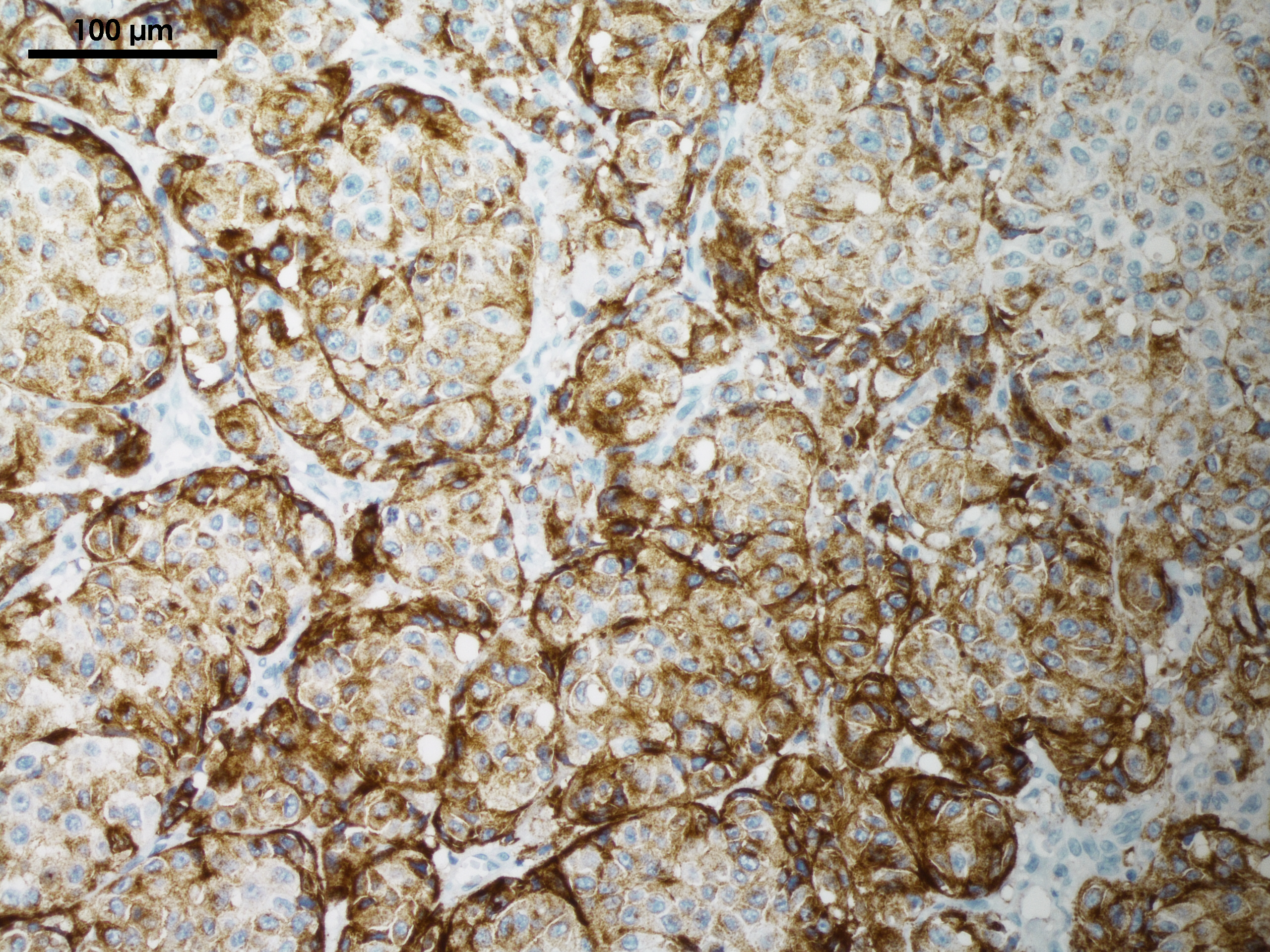

Supplement: Supplementary file 5 — Source data Fig. 2 [file 44321_2025_339_MOESM5_ESM.zip › Figure 2/2C/Mel-57-Met.jpg]

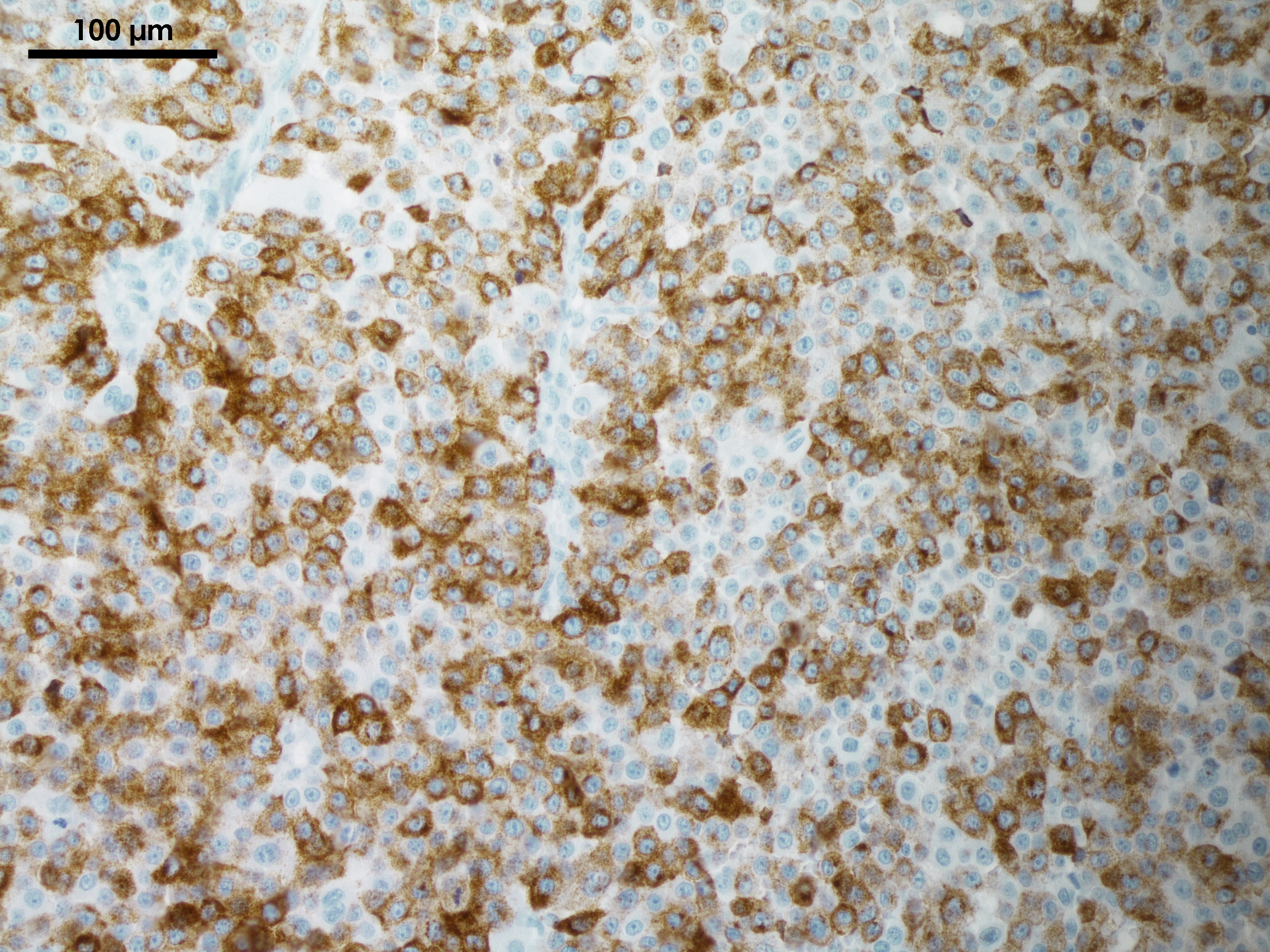

Supplement: Supplementary file 5 — Source data Fig. 2 [file 44321_2025_339_MOESM5_ESM.zip › Figure 2/2C/Mel-57-Patient LN.jpg]

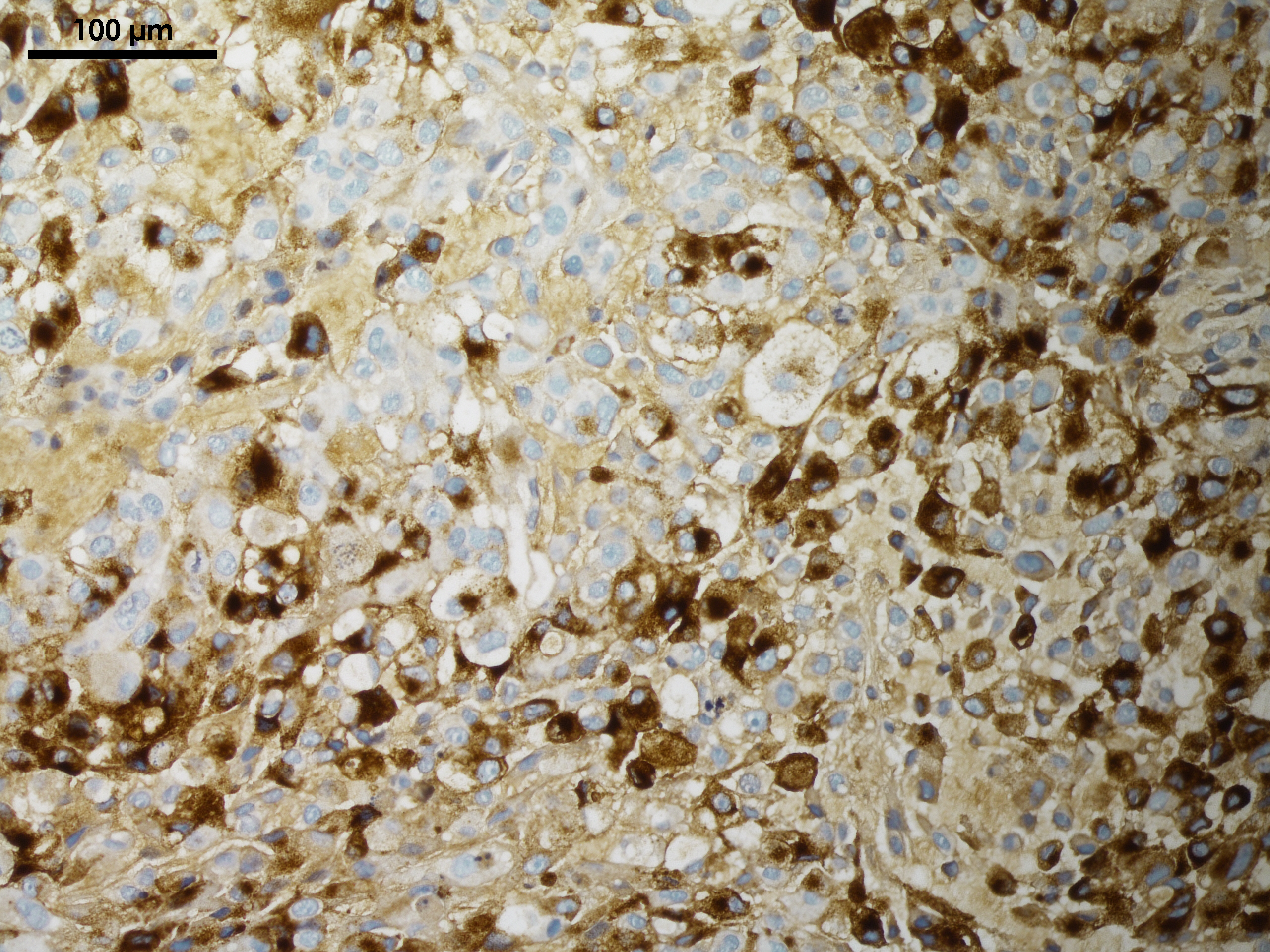

Supplement: Supplementary file 5 — Source data Fig. 2 [file 44321_2025_339_MOESM5_ESM.zip › Figure 2/2C/Mel-57-PDX.jpg]
