# Supplementary material for: Micrometastasis-derived models enable drug testing for early-stage, high-risk melanoma patients
Source: EMBO Mol Med. 2025 Dec 5;18(1):297–324. doi: 10.1038/s44321-025-00339-8 (PMC12808144; doi:10.1038/s44321-025-00339-8)
Supplement: Supplementary file 10 — Expanded View Figures [file 44321_2025_339_MOESM10_ESM.pdf]

Expanded View Figures

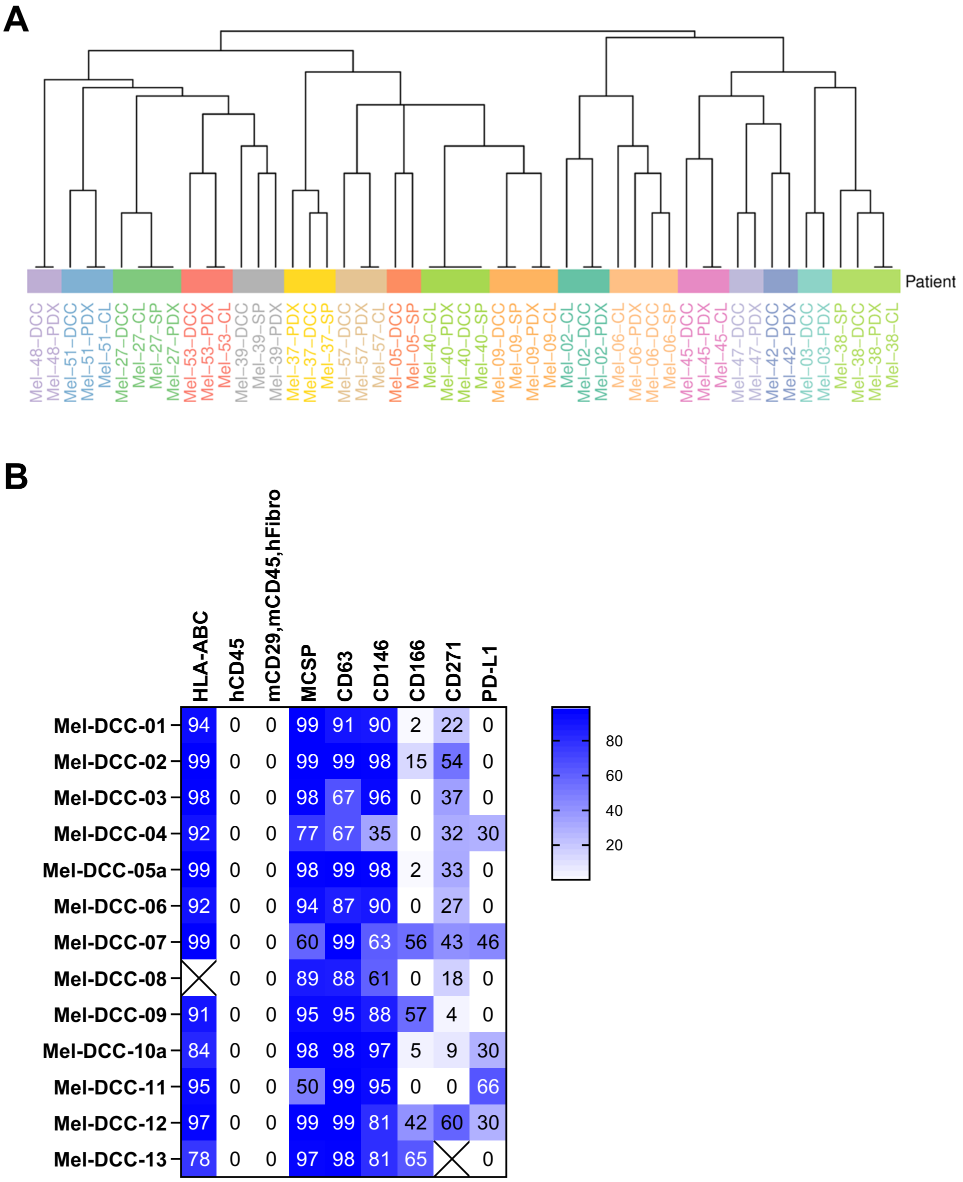

Figure EV1. Sample and Mel-DCC CL characterization.

(**A**) Cluster analysis of LN-DCCs, ex vivo cultured spheres (SP), PDX and CL models based on STR data. Hierarchical sample clustering according to 35 STR features using Euclidean distance and complete linkage. Analysis of Mel-05-PDX failed owing to sample quality issues. (**B**) Flow cytometry analysis of melanoma marker (MCSP, CD63, CD146, CD166, CD271) and immune checkpoint ligand (PD-L1) expression on Mel-DCC CLs. The human origin was verified using an anti-human HLA-ABC antibody. To exclude contaminations and ensure purity, CLs were tested for non-melanoma human cells (CD45, fibroblasts) and mouse cells (CD29, CD45). The scores (color scale) indicate the percentage of marker-positive cells compared to isotype controls.

© The Author(s)

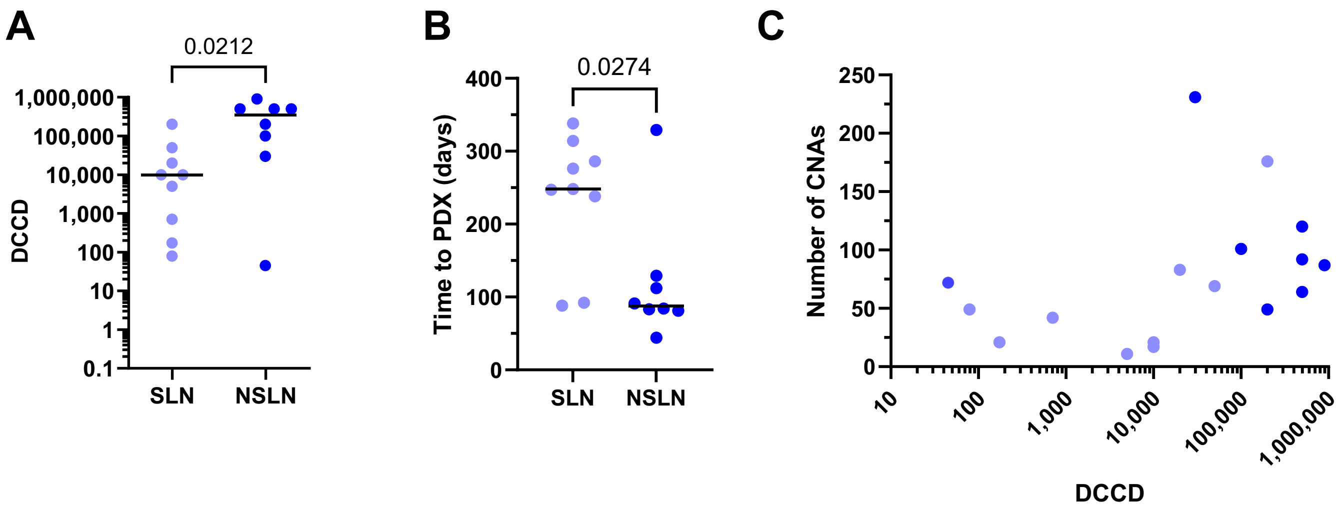

Figure EV2. Association of DCCD and LN type, time to PDX generation and number of aberration calls in PDX.

(**A**) Comparison of DCCD of SLNs and NSLNs that successfully formed PDXs by approach I (SLNs: $n = 9$ patients, NSLNs: $n = 8$ patients). Each dot represents an individual patient, the horizontal line indicates the median. *P* value according to an unpaired, nonparametric Mann–Whitney test; $^*P = 0.0212$. Light and dark blue indicate SLNs and NSLNs, respectively. (**B**) Comparison of the time needed for PDX generation (time from sample receipt to tumor harvest) for SLNs and NSLNs. The analysis included the fastest available model for each patient. Each dot represents an individual patient, the horizontal line indicates the median. *P* value according to an unpaired, nonparametric Mann–Whitney test; ns, $P = 0.0274$). Light and dark blue indicate SLNs and NSLNs, respectively. (**C**) Correlation of DCCD and number of CNAs. Spearman *r* correlation, $r = 0.5603$; $^*P = 0.0210$. Each plot represents an patient, the horizontal line indicates the median. Light and dark blue indicate SLNs and NSLNs, respectively.

© The Author(s)

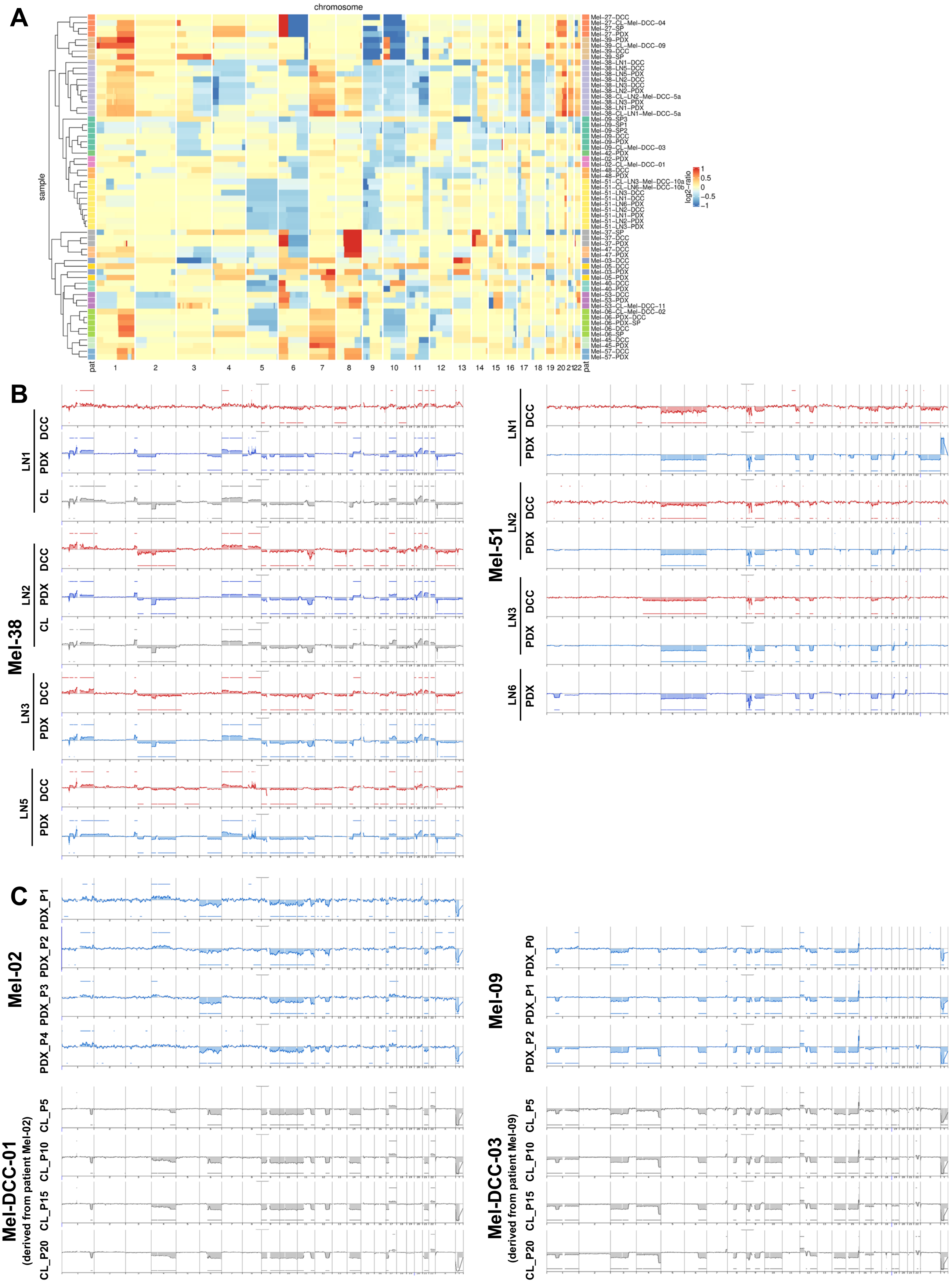
A
chromosome
sample
log2-ratio
pat
B
Mel-38
LN1
LN2
LN3
LN5
DCC
PDX
CL
Mel-51
LN6
C
Mel-02
PDX_P1
PDX_P2
PDX_P3
PDX_P4
Mel-09
PDX_P0
Mel-DCC-01
(derived from patient Mel-02)
Mel-DCC-03
(derived from patient Mel-09)
CL_P5
CL_P10
CL_P15
CL_P20

Figure EV3. Genome-wide CNA profiles of DCC-derived xenograft models (PDX), matched pairs of DCCs, DCC-derived spheres (SP) or in vitro-generated cell lines (CL).

(**A**) Clustering of log_2-fluorescence ratios of 4407 chromosomal bins using Euclidean distance and complete linkage. Negative log_2 ratios indicate genomic losses, positive log_2 ratios genomic gains relative to the median copy number. (**B**) CNA profiles of 2 patients, from which multiple LNs and corresponding models were obtained. (**C**) CNA profiles of PDX and CL models derived from patient Mel-02 and Mel-09. Serial in vivo/in vitro passages are displayed, respectively.

© The Author(s)

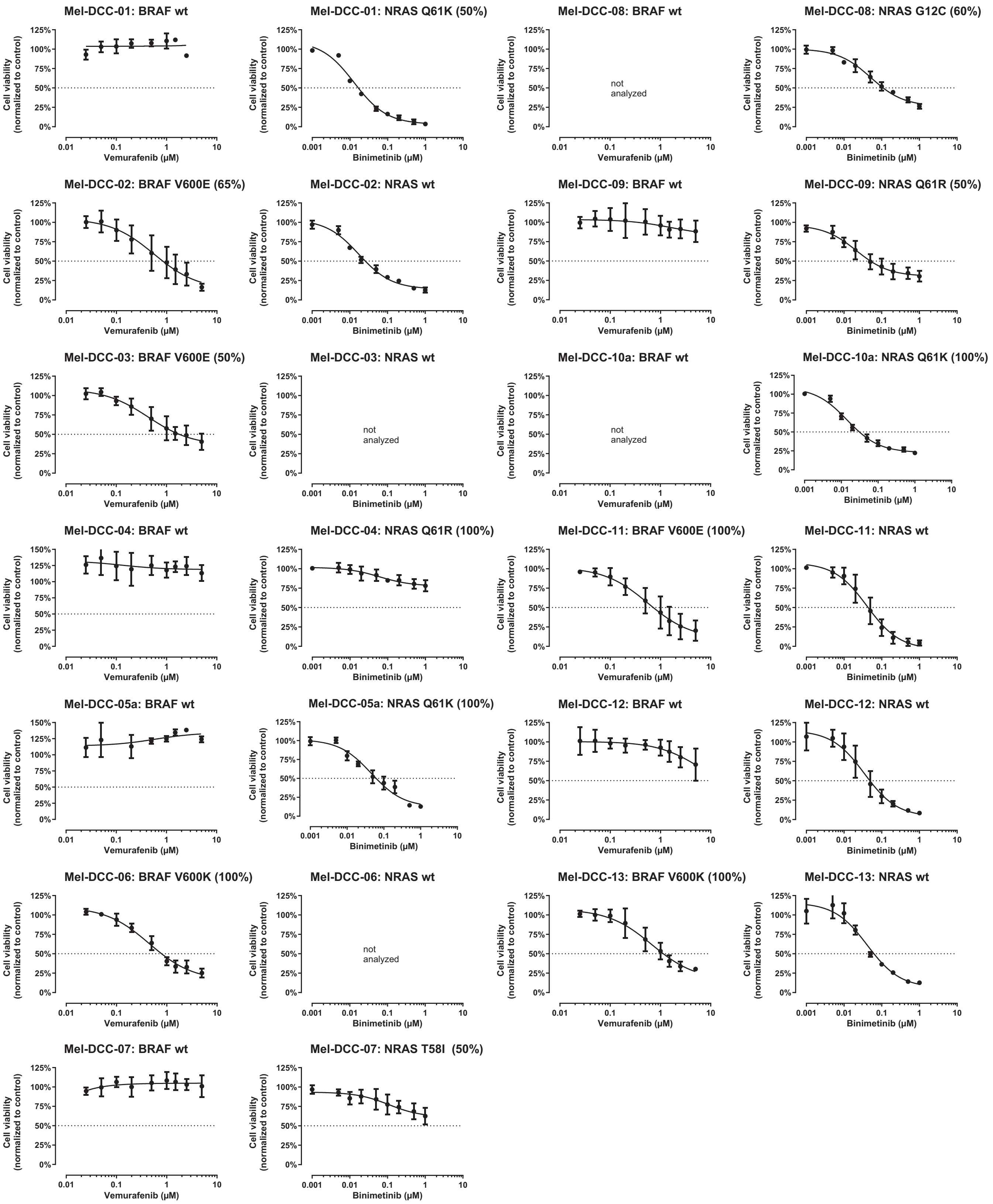
Mel-DCC-01: BRAF wt
Mel-DCC-01: NRAS Q61K (50%)
Mel-DCC-08: BRAF wt
not analyzed
Mel-DCC-08: NRAS G12C (60%)
Mel-DCC-02: BRAF V600E (65%)
Mel-DCC-02: NRAS wt
Mel-DCC-09: BRAF wt
Mel-DCC-09: NRAS Q61R (50%)
Mel-DCC-03: BRAF V600E (50%)
Mel-DCC-03: NRAS wt
not analyzed
Mel-DCC-10a: BRAF wt
not analyzed
Mel-DCC-10a: NRAS Q61K (100%)
Mel-DCC-04: BRAF wt
Mel-DCC-04: NRAS Q61R (100%)
Mel-DCC-11: BRAF V600E (100%)
Mel-DCC-11: NRAS wt
Mel-DCC-05a: BRAF wt
Mel-DCC-05a: NRAS Q61K (100%)
Mel-DCC-12: BRAF wt
Mel-DCC-12: NRAS wt
Mel-DCC-06: BRAF V600K (100%)
Mel-DCC-06: NRAS wt
not analyzed
Mel-DCC-13: BRAF V600K (100%)
Mel-DCC-13: NRAS wt
Mel-DCC-07: BRAF wt
Mel-DCC-07: NRAS T58I (50%)
Cell viability (normalized to control)
Vemurafenib (µM)
Binimetinib (µM)
0%
25%
50%
75%
100%
125%
150%
0.001
0.01
0.1
1
10

 © The Author(s)

◀ **Figure EV4. Dose response to targeted therapies in Mel-DCC CLs.**

Mel-DCC CLs were incubated for 5 days with doses ranging from 0.025 µM to 5 µM for Vemurafenib and from 0.001 µM to 1 µM for Binimetinib. Cell viability was monitored with CellTiter-Blue®. Dots indicate mean values ± SD of three biological replicates. Variant allele frequency is indicated in parentheses. Gray dotted lines indicate the IC_{50} values (also given in Table 2).

© The Author(s)

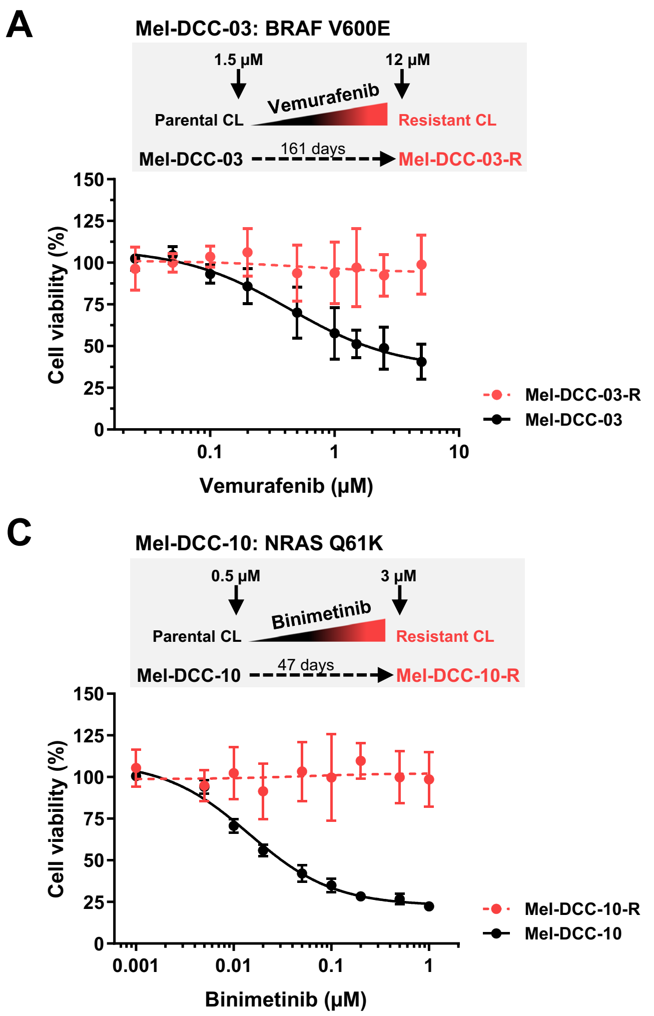

Figure EV5. Generation of resistant Mel-DCC CLs.

(**A**) Generation of a Vemurafenib-resistant BRAF V600E-mutated line (Mel-DCC-03-R). The resistant line (red) was generated through stepwise exposure of the parental line (Mel-DCC-03, black) to increasing concentrations of Vemurafenib over the indicated timeframe. Each dot indicates the mean ± SD of biological replicates (Mel-DCC-03, $n = 4$; Mel-DCC-03-R, $n = 5$). (**B**, **C**) Generation of Binimetinib-resistant NRAS-mutated melanoma cell lines. Sensitivity of Mel-DCC-05 vs. Mel-DCC-05-R (**B**) and Mel-DCC-10 vs. Mel-DCC-10-R (**C**) to Binimetinib are shown. Resistant lines (red) were generated through stepwise exposure of parental lines (black) to increasing concentrations of Binimetinib over the indicated timeframe. Each dot indicates the mean ± SD of biological replicates (Mel-DCC-05 and -10, $n = 4$; Mel-DCC-05-R and -10-R, $n = 5$).

 © The Author(s)
